# Supplementary material for: Ubiquinone Metabolism and Transcription HIF-1 Targets Pathway Are Toxicity Signature Pathways Present in Extracellular Vesicles of Paraquat-Exposed Human Brain Microvascular Endothelial Cells
Source: Int J Mol Sci. 2021 May 11;22(10):5065. doi: 10.3390/ijms22105065 (PMC8150401; doi:10.3390/ijms22105065)
Supplement: Supplementary file 1 [file ijms-22-05065-s001.zip › ijms-1210631-supplementary/Figures_S1_S2_S3.pdf]

Figure S1 MTS and LDH assay on HBMECs after paraquat exposure

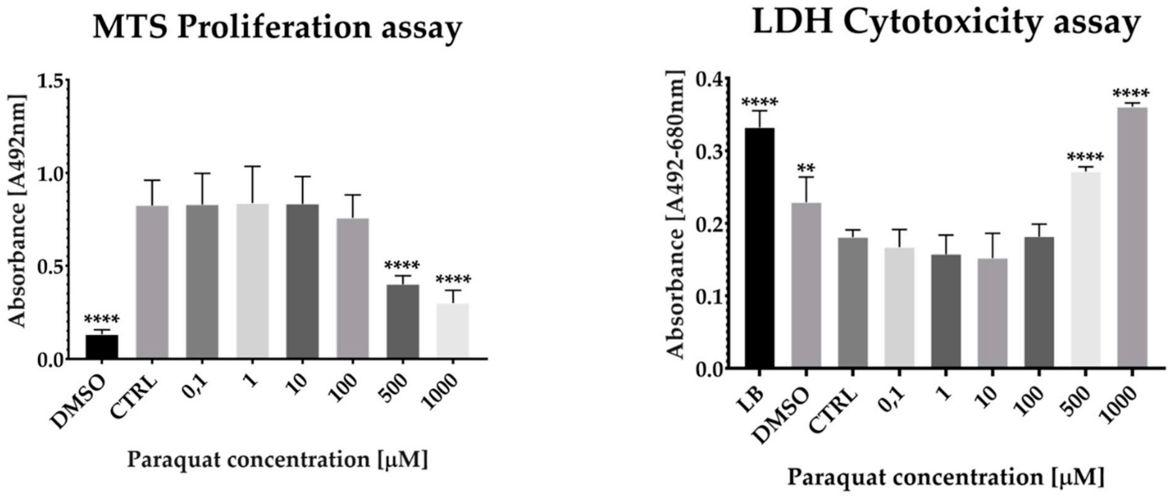

Figure S1. Effect of 24h-exposure to PQ at different concentrations (0,1, 1, 10, 100, 500 and 1000 μM) on viability and cell death assessed using MTS and LDH assays, respectively. Data are represented as means ±SD of six biological replicates. ** corresponds to a *p*-value ≤ 0,01 and **** corresponds to a *p*-value ≤ 0,0001.

Figure S2 Volcano plot of EV proteins after paraquat exposure

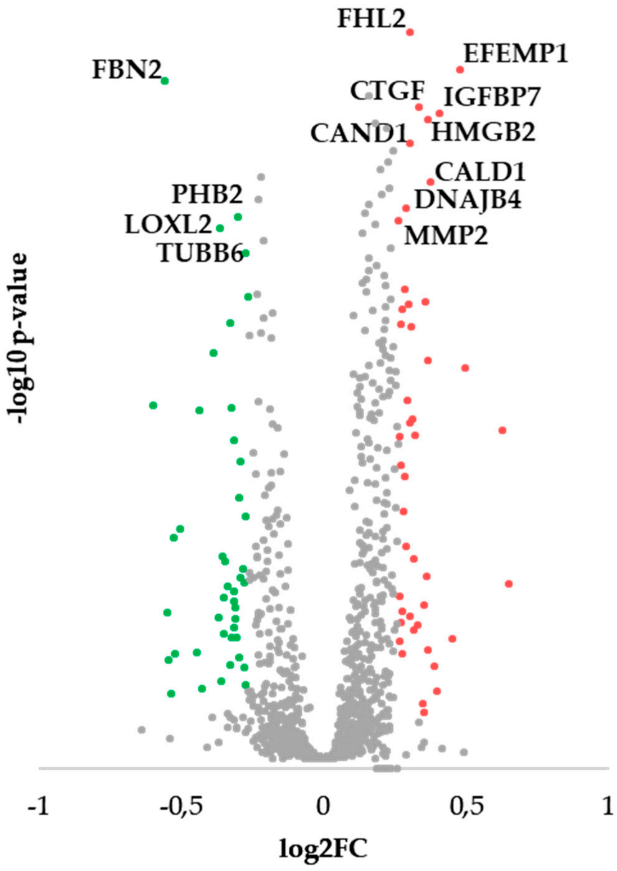

Figure S2. Volcano plots displaying the distribution of all proteins after PQ treatment at 100 µM with relative protein abundance $\log_2$(PQ 100 µM/control ratio) plotted against its significance level $-(\log_{10})$ *p*-value, showing significantly ($P \leq 0.05$) increased (PQ 100 µM/control ratio > 1.2; red) and decreased (PQ 100 µM/control ratio ≤ 0.83; green) EV proteins after PQ treatment.

Figure S3 Biological process gene ontology enrichment of changing EV proteins after paraquat exposure

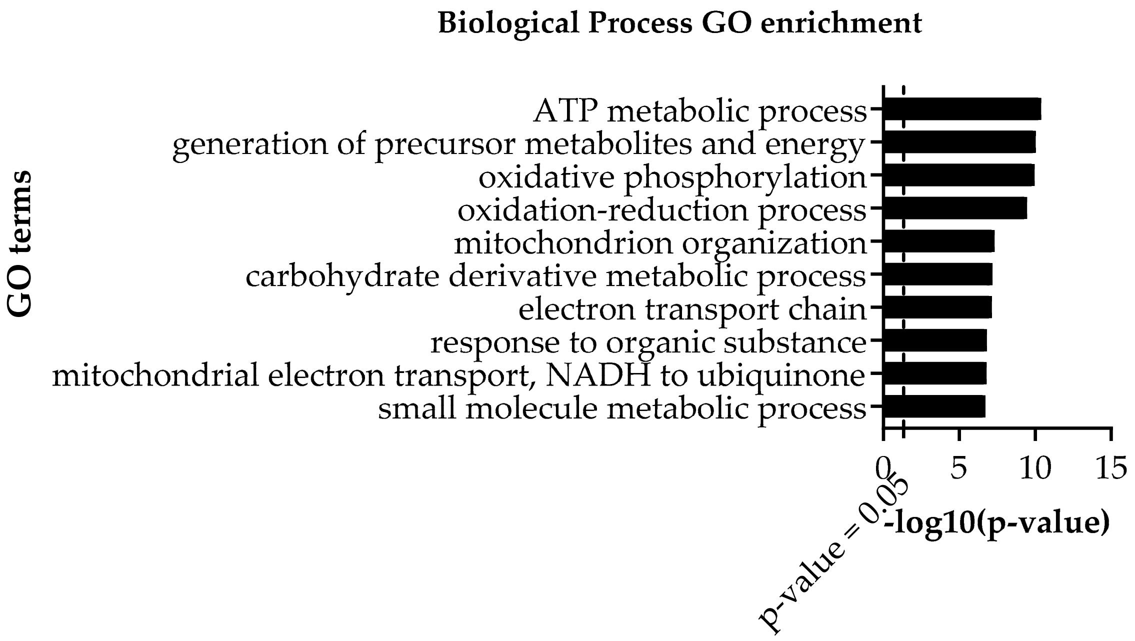

Figure S3. Top ten enriched biological process GO enrichment by MetaCore™ software for the lists of changing proteins (FC > 1.2, p-value ≤ 0.05, N = 3) after PQ treatment on EVs from HMBECs. X axis corresponds to −log10(p-value), Y axis corresponds to the GO terms and the dashed line represents the p-value cut-off of 0.05.
